# Verification of a theory of planned behavior model of medication adherence in Korean adults: focused on moderating effects of optimistic or present bias

**DOI:** 10.1186/s12889-021-11460-x

**Published:** 2021-07-13

**Authors:** Kyung Hyun Suh

**Affiliations:** Department of Counseling Psychology, Sahmyook University, #227 International Education Building, 815 Hwarang-ro, Nowon-gu, Seoul, 01795 South Korea

**Keywords:** Adherence, Attitude, Perceived behavioral control, Optimistic bias, Present bias

## Abstract

**Background:**

To prevent or recover from a disease, the prescriptions for medications must be correct, and the patient must comply with the medication’s instructions. Therefore, this study verified the theory of planned behavior (TPB) model to predict medication adherence among Korean adults and examined the role of optimistic or present bias in that model.

**Methods:**

The participants were 357 Korean male and female adults whose ages ranged from 18 to 76 (*M* = 41.53, *SD* = 9.89). Their medication adherence was measured with the Morisky Green Levine Scale. The study examined TPB factors with modified items related to medication adherence and optimistic bias with items developed based on the concept and on previous studies.

**Results:**

An alternative TPB model, including a direct path from attitude to behavior, a direct path from the perceived behavioral control to the behavior, and an insignificant path from behavioral intention to behavior, was validated for Korean adults’ medication adherence. This model was found to be moderated by optimistic or present bias.

**Conclusions:**

The findings of this study should provide useful information for future research and for medical or health professionals who wish to improve the medication adherence of their patients.

## Background

Koreans’ interest in health has been heightened in the twenty-first century as their living standards have improved and their life expectancy has increased significantly [1]. Because of their high level of interest in health, it may seem likely that they intend to practice health behaviors to improve, maintain, or recover their health, but in fact, it is reported that they do not do so. Even when they have a prescription from a medical care provider or health professional, Koreans often do not, in actuality, adhere to the prescription instructions in practice. In a meta-analysis of over 500 studies, DiMatteo [2] concluded that the proportion of people who do not adhere to prescription drugs for life-threatening diseases is around 25%. In addition, medication adherence rates reported in studies may be inflated [3]. In a report by the Korea Institute for Health and Social Affairs (KIHASA) [4], which studied Koreans’ adherence to the treatment of chronic diseases, the medication adherence ratio based on the self-report adherence scale was not exceptionally low. However, the rate at which study participants were prescribed and purchased the drug was significantly lower than the self-report ratio. Park and colleagues [4] also conducted a Focus Group Interview (FGI), and confirmed that the actual rate may be much lower than the rate of self-reported medication adherence.

For recovery from or prevention of disease, prescriptions need to be correct. However, even when the prescription is appropriate, treatment or prevention cannot succeed if the patient does not adhere to it [3]. Adherence or compliance is, therefore, essential to disease treatment or health promotion. Thus, in this study, I sought to verify models that can predict prescription adherence in terms of public health and to explore variables that moderate the model.

Many researchers have attempted to explain health-risk behaviors or non-adherence with health behaviors through the health belief model [5], the theory of reasoned action [6], self-efficacy [7], the theory of planned behavior [8], the precaution adoption process model [9], and the trans-theoretical model [10]. In this study, I used the theory of the planned behavior (TPB) as a theoretical model to predict medication adherence. TPB has been applied to explain various health behaviors or health-risk behaviors, including underage drinking [11], the health promoting behaviors of chronic pain patients [12], risk behaviors that can cause coronary heart disease [13], smoking [14], physical activity [15], cancer screening [16], and healthy eating habits [17].

TPB [8] includes attitudes toward health behavior, subjective norms, perceived behavioral control, and behavioral intention. This adds perceived behavioral control to the theory of reasoned action [6], which was developed because attitudes are limited in predicting behavior. In theory, behavioral intention accounts for most of the variance of behavior, and attitudes and subjective norms can affect behavior. Behavioral intention refers to an aim to carry out a certain behavior, and may be the closest antecedent of the behavior. As the most determinant variable of behavior, it may be the channel through which attitude, subjective norms, and perceived behavioral control influence a given behavior [18].

TPB is also related to social cognitive theory [19] because perceived behavioral control is nearly the same concept as self-efficacy in social cognitive theory. Many studies [20] have shown that perceived behavioral control accounts most for the variance of behavior in the TPB model. Ajzen [8] also considered models with a direct path from perceived behavioral control to behavior (p. 182). Therefore, this study compares the goodness-of-fit of alternative models with direct routes and with a model with no direct path between attitude or perceived behavioral control and medication adherence behavior. In addition, because attitudes include behavioral factors as well as affective and cognitive factors [21], there may be a direct impact of attitude on adherence to certain behaviors; it is thus necessary to verify models that include a direct path from attitude to adherence behavior.

In this study, optimistic bias was adopted as a variable that might affect adherence. Optimistic bias refers to one’s vague positive expectation that he or she is less likely to be at risk than others [9]. Weinstein [9] assumed that optimistic bias plays a role in adopting disease-preventing behavior and included it as a psychological variable in the precaution adoption process model for health behavior. Although it has not been extensively studied in Korea, some studies [22, 23] have found that optimistic bias may influence health behaviors.

Optimistic bias may be an internal and personal factor that also could be applied to other areas as well as health behaviors, O’Sullivan [24] believed it was a prevalent personal characteristic regardless of gender, age, and race. Furthermore, some researchers argue that animals such as rats and birds also have an optimistic bias [25]. In addition, optimistic bias was shown in both positive and negative situations, but the intensity was stronger in negative situations [26]. This cognitive bias can cause great damage to individuals if it operates in a dangerous situation that could result in major injuries or fatal diseases. Some empirical studies found that optimistic bias was highly likely to lead to actions exposing the individual to the risk of crime [27], the risk of stock investment failure [28], and the risk of bungee jumping injury [29]. A previous studies [22, 23, 30] have shown that optimistic bias is a meaningful factor in disease-preventing behavior, this study assumes that it could be a moderator in the TPB model as well as the precaution adoption process model for predicting medication adherence.

In addition, present bias was also adopted as a variable that could affect the model predicting medication adherence. Present bias, also called delay discounting or time preference, is a variable studied with delayed gratification. Delay discounting, which is an economic term, refers to the relative value of a later time, as opposed to if it were readily available [31]. In other words, if the expected benefits or outcomes are not quickly achieved or are delayed, the value will be discounted. This delay discounting is due to a bias that places greater importance on the present and undervalues future outcomes.

Such delay discounting is also found in practicing health risk behavior and health behavior. For example, Fields, Ramos, and Reynolds [32] found that delay discounting was likely to lead to underestimating the consequences of a health risk behavior, leading to the action being carried out. A smoking study [33] found that either present bias or delay discounting could moderate the relationship between impulsivity and smoking behaviors, and if the effects of exercise appear after a long period of time, no matter how positive the effect, the incentive could be reduced [34]. Based on previous studies, it can be assumed that individuals are less likely to take medication if they recognize delay discounting in the medication’s effects. However, some researchers [35] point out that this present bias or delay discounting may vary depending on culture; in this study, it was posited to be a universal personal tendency, and it was assumed that present bias also related to medication adherence and affects the TPB model, which predicts medication adherence.

**Fig. 1:**
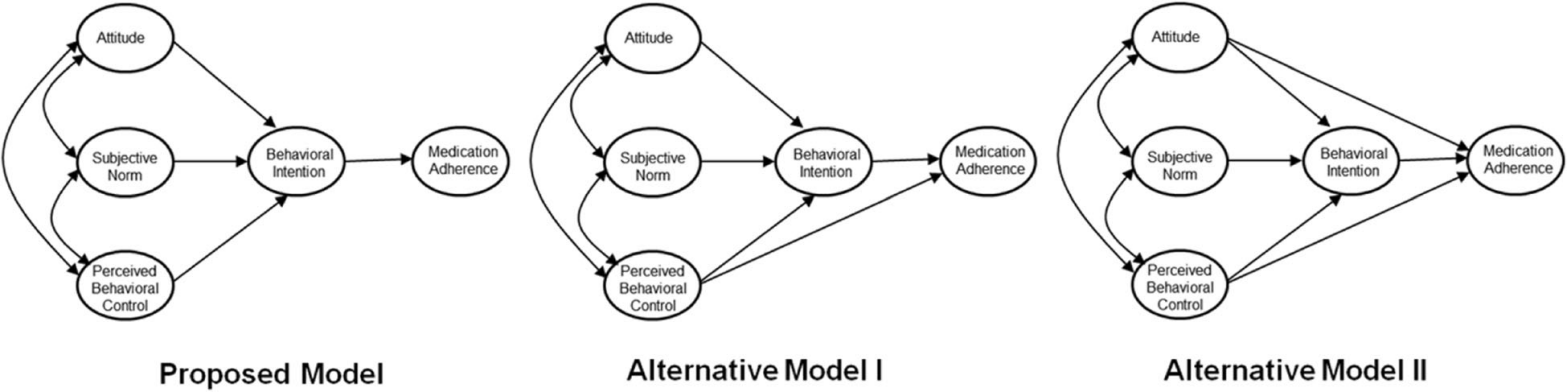
Proposed and Alternative Models of TPB for Medication Adherence

This study designed and examined a TPB model to predict Koreans’ medication adherence and analyzed whether the model was moderated by optimistic or present bias. First, this study verified a proposed model that does not include a direct path between attitude or perceived behavioral control and medication adherence behavior, and compared it with alternative models that include direct paths. Alternative model I includes only the direct path between perceived behavioral control and medication adherence behavior, while interpersonal model II adds the direct path between attitude and medication adherence to the model (Fig. 1). Next, whether optimistic bias or present bias moderated the model adopted in this study was investigated. This analysis is expected to provide useful information to promote Koreans’ medication adherence.

## Methods

### Participants

The participants in the study were 357 Korean male and female adults aged 18 or older selected using convenience sampling. Data were collected with a questionnaire and informed consent posted on an Internet portal site (NAVER). The study was promoted on NAVER BAND and internet communities. Ordinary Korean adults who look for information on the Internet and who have meetings on social networking sites would have had the opportunity to see these promotion and participate in the survey. Participants’ ages ranged from 18 to 76, and their average age was 41.94 (*SD* = 9.89). Their data were collected mainly through the Internet, though some were collected directly through research assistants. As no effort was made to match the gender ratio in the process of gathering data through the Internet, the proportion of women was 67.2%. The gender ratio difference was not likely to affect the study because gender differences were only found with present bias (*p* < 0.01); no gender differences were found in other variables.

The educational background of the participants was 218 college graduates (61.1%), 95 high school graduates (26.6%), 41 graduate graduates (11.5%), two middle school graduates (0.6%), and one who did not report their academic background (0.3%). Most respondents (90.2%) reported their family economic status as moderate, while 35 respondents (9.8%) reported theirs as low.

Moreover, 68 (19.0%) of participants self-identified as smokers, and 121 (33.9%) reported that they did not drink at all, while 56 (15.7%) reported that their average alcohol consumption was one or two standard drinks, 69 (19.3%) reported that theirs was four drinks, and 111 (31.1%) reported consuming more than seven drinks at a time on average.

### Instruments

#### Factors of TPB

To measure the factors of the TPB model associated with participants’ medication adherence, questions based on Ajzen’s theory [36] and studies conducted in Korea [37] were modified to suit the subject matter of this study. Items for attitude concern whether participants think positively or negatively in regard to medication adherence (e.g., “Even if the symptoms have improved a little, it is beneficial to continue taking medication as prescribed.”), while items for subjective norms concern how they perceive the social norms of medication adherence (e.g., “People around me would want me to take the prescribed medicine”), and items of perceived behavioral control concern how much control they exhibit when taking medicine (e.g. “I have the self-control to keep taking the medicine prescribed by the doctor”). In addition, items of behavior intention concern medication compliance (e.g., “I intend to take the medicine as prescribed”). Each factor was measured with four items, and each item was scored on a seven-point Likert scale ranging from 1 (*not at all*) to 7 (*always*). The internal consistencies (Cronbach’s α) of factors of the TPB model were 0.85 for attitude, 0.84 for subjective norms, 0.89 for perceived behavioral control, and 0.95 for behavior intention in this study.

#### Medication adherence

The participants’ behavior in relation to medication adherence was measured using the Morisky Green Levine Scale (MGLS) [38]. This scale was developed to measure how well hypertension patients complied with the prescribed antihypertensive drug. In this study, participants were asked about compliance with or adherence to all prescription drugs in addition to antihypertensive medication. This scale consists of four items, and uses a dichotomous question to assess medication adherence, but a seven-point rating scale was used in this study to maximize the variance. The translated items were taken from previous studies using the MGLS [39]. The internal consistency (Cronbach’s α) was 0.62 in this study, which was somewhat low, because the number of questions was relatively small.

#### Optimistic bias

The participants’ optimistic bias against the results of medication noncompliance and unhealthy behavior was measured using modified items from a questionnaire developed by Suh and colleagues [22] based on Weinstein’s two questions for measuring optimistic bias [40]. The items were modified to suit the subject matter of this study: “Other people may suffer serious consequences if they do not take prescribed drugs, but there will not be a big problem if I do not do so” and “Even if other people have health problems because they do not practice their health behaviors, I will not have such problems.” This questionnaire consists of six items, and each item was scored on a seven-point Likert scale ranging from 1 (*not at all*) to 7 (*always*). The internal consistency (Cronbach’s α) was 0.88 in this study. In factor analysis with Principal Axis Factoring (PAF) and orthogonal varimax rotation, a factor was retained.

#### Present bias

To measure present bias toward medicinal effect and healthy behavior, a questionnaire developed by Suh was used [41]. This questionnaire measures how much less valuable participants perceive the effects of medication adherence or health behavior due to delays. Present bias is typically measured with a condition that provides a delay discounting task [42], but due to the nature of this study, the researcher used self-report questions focused on to what degree participants recognized delayed discounts regarding the effects of medication adherence and healthy behaviors. Examples of the items are, “I think exercising will have too late of a health effect,” and “Generally, even if a prescribed drug is taken, the effect is delayed.” The questionnaire comprises six items rated on a scale ranging from 1 (*not at all*) to 4 (*always*). The internal consistency (Cronbach’s α) was 0.95 in this study. A factor was retained for factor analysis with these six items.

### Data analysis

IBM SPSS Statistics for Windows and AMOS (Analysis of Moment Structure) were used for all the statistical analyses; Pearson-Product Moment correlational analysis and t-tests to check for gender differences were performed with SPSS, and path analysis and analysis of moderating effects were performed with AMOS. For these parametric statistical analyses, it was necessary to check the mean and standard deviation, skewness, and kurtosis of the data.

Maximum Likelihood (ML) estimate was used to estimate the model when performing path analysis with AMOS. The goodness-of-fit evaluation was performed using the relative goodness-of-fit indices NNFI (Non-normed Fit Index) and CFI (Comparative Fit Index) as well as the absolute goodness-of-fit indices GFI (Goodness of Fit Index) and RMSEA (Root Mean Square Error of Approximation). In general, an RMSEA value of < 0.05 is considered a ‘close fit,’ while < 0.08 suggests a reasonable model fit and < 0.10 indicates an acceptable model fit. Furthermore, a GFI and CFI larger than 0.90 and an NNFI larger than 0.95 suggests a relatively good model fit. An NNFI of > 0.90 and < 0.95 indicates an acceptable model fit.

Finally, bootstrapping was used to examine the causal relationships of variables and analyze the significance of the mediated effect. In addition, the TPB model with moderating effects used individual parameter verification to estimate the moderating effect from a single model of two samples. Moreover, verification of the model with moderating effects was performed by estimating the moderating effect from a single model of two samples, that is, verification of individual parameter differences. When it was unclear whether to conclude that there was a moderating effect, analysis of the differences in two models of two samples was also used. Groups with high and those with low optimistic bias and present bias were divided by the medians of 14 and 27, respectively. Therefore, those who belong to the high optimistic bias group were participants who scored 15 or more on the questionnaire, and those who belonged to the high present bias group were participants who scored 28 or more on the items.

## Results

### Relationships between variables involved in medication adherence

The results of the analysis of the relationships among the variables from TPB, optimistic bias, present bias, and medication adherence are shown in Table 1. First, the skewedness and kurtosis were checked and the absolute value did not exceed 1 for any of the variables, indicating that variances of each variable were close to a normal distribution.

**Table 1:** Correlational matrix of factors of TPB, optimistic bias, and present bias for medication adherence (*N* = 357)

Variables	1	2	3	4	5	6	7
**1.** Attitude							
**2.** Subjective norm	0.56^***^						
**3.** Perceived Behavioral Control	0.52^***^	0.52^***^					
**4.** Behavioral Intention	0.67^***^	0.54^***^	0.74^***^				
**5.** Medication Adherence	0.34^***^	0.18^***^	0.35^***^	0.34^***^			
**6**. Optimistic Bias	−0.27^***^	−0.12^*^	− 0.24^***^	−0.28^***^	− 0.04		.
**7.** Present Bias	0.02	0.05	−0.06	0.02	−0.19^***^	0.13^*^	
**Mean Standard Deviation**	21.23 (4.06)	20.12 (4.17)	21.65 (4.16)	22.00 (4.14)	14.79 (4.56)	15.08 (6.66)	27.08 (5.95)
**Skewedness**	−0.44	−0.25	− 0.53	−0.56	0.20	0.65	−0.21
**Kurtosis**	0.29	−0.26	0.22	0.53	−0.26	0.11	0.04

^*^ *p* < 0.05, ^**^p < 0.01, ^***^p < 0.001

The correlation analysis found significant positive correlations among the TPB variables involved in medication adherence. In particular, attitudes (*r* = 0.67, *p* < 0.001) and perceived behavioral control *(r* = 0.74, *p* < 0. 001) were closely related to the behavioral intention of medication adherence, accounting for 44.9 and 54.8% of the variance of behavioral intention of medication adherence, respectively.

On the other hand, there was no significant correlation between optimistic bias and medication adherence (*r* = − 0.04, *n.s.*), though optimistic bias was negatively correlated with attitude (*r* = − 0.27, *p* < 0.001), subjective norms (*r* = − 0.12, *p* < 0.05), perceived behavioral control (*r* = − 0.24, *p* < 0.01), and behavioral intention of medication adherence (*r* = −.28, *p* < 0.001). The present bias for medicinal effect was significantly correlated with the medication adherence (*r* = − 0.19, *p* < 0.001), but it was not significantly correlated with the TPB variables involved in medication adherence.

### Path analysis of the proposed model and alternative models

This study presented a proposed model based on the TPB and attempted to determine the optimal model by comparing its goodness of fit with that of alternative model I, which added the path of perceived behavioral control → medication adherence, and of alternative model II, which added the path of attitude → medication adherence as well as perceived behavioral control → medication adherence. Based on both accountability and simplicity, the fit index used in this study was in addition to the commonly used NNFI and RMSEA, as well as GFI and CFI. .

The χ^2^ value of the proposed model was 34.25 (*df* = 3, *p* < 0.001), and the goodness-of-fit index was GFI = 0.965, NNFI = 0.856, CFI = 0.957, and RMSEA = 0.120 (Table 2). A significant χ^2^ value suggested that this model may vary depending on the number of samples. The GFI and CFI values were found to be above 0.90 and fell within the range of good model conditions, but the NNFI (below 0.90) and RMSEA values (above 0.10) were outside the values for good model conditions.

**Table 2:** Comparison of the goodness of fit between the proposed model and alternative models

Model	χ^**2**^	*df*	GFI	NNFI	CFI	RMSEA (90% confidence interval)
Proposed Model	18.78^***^	3	0.980	0.929	0.978	0.122 (0.173 ~ 0.177)
Alternative Model I	11.11^**^	2	0.988	0.937	0.987	0.113 (0.055 ~ .0182)
Alternative Model II	3.18	1	0.996	0.970	0.997	.078 (0.000 ~ 0.182)

^**^p < 0.01, ^***^p < 0.001

The χ^2^ value of alternative model I with the added path of perceived behavioral control → medication adherence was 11.11 (*df* = 2, *p* < 0.01), and the goodness-of-fit indices were GFI = 0.988, NNFI = 0.937, CFI = 0.987, and RMSEA = 0.113 (Table 2). The significant χ^2^ value for the proposed model suggested that it may vary depending on the number of samples. The GFI and CFI values were shown to be above 0.90 and indicated a good model. NNFI value was acceptable, but the RMSEA values (above 0.10) were outside the range for good model conditions, similar to the proposed model.

On the other hand, the χ^2^ value of alternative model II with the added paths of perceived attitude → medication adherence and perceived behavioral control → medication adherence was 3.18 (*df* = 1, *n.s.*) and the goodness-of-fit indices were GFI = 0.996, NNFI = 0.970, CFI = 0.996, and RMSEA = 0.078 (Table 2). First, the fact that the χ^2^ value was not significant means that the model represented the total population of this sample well, so there was no statistically significant difference between the observed and estimated matrices, that is, the covariance matrix. The GFI, NNFI, and CFI values met the conditions for a good model, all over 0.90. It was also found that the RMSEA value was less than 0.08, also indicating a good model. This means that there was no need to compare the goodness-of-fit with χ^2^ differentiation based on a nested relationship, suggesting that alternative model II should be adopted. In other words, this study validated the model with the addition of the attitudes → behavior path and perceived behavioral control → behavior path as a useful theory of planned behavior for medication adherence.

**Fig. 2:**
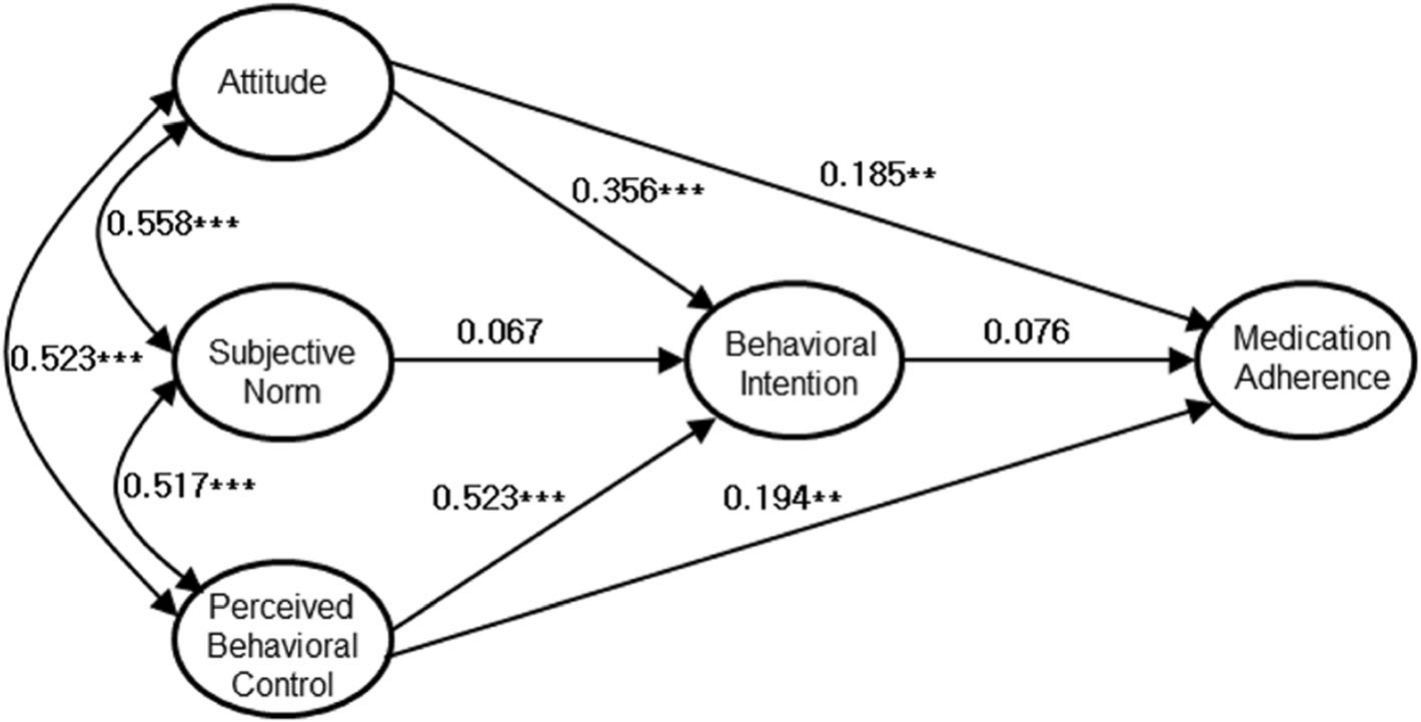
Path map of alternative model II of TPB for medication adherence (^**^p < 0.01, ^***^p < 0.001)

The path coefficients in alternative model II in this study are shown in Fig. 2 and Table 3. For each path coefficient in the adoption model involved in medication adherence, the results showed that the more positive the attitude, the more likely there is to be intention of medication adherence (*β* = 0.356, *p* < 0.001), and the higher the level of perceived behavioral control, the stronger the intention of medication adherence is (*β* = 0.523, *p* < 0.001). In addition, if people had a positive attitude toward medication, they were more directly compliant with the prescription (*β* = 0.185*, p* < 0.01), and if they perceived themselves as able to control their medication behavior, they were better at taking medication, *β* = 0.195, *p* < 0.01. However, the path between the intention and behavior of medication adherence was not significant.

**Table 3:** Estimated parameter values of adopted model (alternative model II) for medication adherence

Predictors	Non-Standardized Weight	Standardized Weight	S.E.	C.R.
Attitude → Intention	0.363	0.365	0.041	9.06^***^
Subjective norm → Intention	0.066	0.067	0.039	1.71^†^
Perceived behavioral control → Intention	0.521	0.523	0.038	13.73^***^
Attitude → Adherence	0.208	0.185	0.073	2.83^**^
Perceived behavioral control → Adherence	0.213	0.195	0.080	2.66^**^
Intention → Adherence	0.083	0.076	0.092	0.91

^†^ *p* < 0.10, ^*^p < 0.05, ^**^p < 0.01, ^***^p < 0.001

Alternative model II was adopted because the direct paths of attitude to medication adherence and perceived behavioral control to medication adherence behavior were significant. However, the results of the analysis of the mediated effects (Table 4) showed that the indirect paths from attitude through intention, from subjective norms through intention, and from perceived behavioral control through intention to medication adherence were not significant. This is because the relationship between behavioral intention and behavior was not significant in this model.

**Table 4:** Mediating effects of the adopted model (alternative model II) for medication adherence

Predictors	Non-Standardized Effect	Standardized Effect
Attitude → Intention → Adherence	0.363	0.027
Subjective norm → Intention → Adherence	0.006	0.005
Perceived behavioral control → Intention → Adherence	0.043	0.040

### Moderating effect of optimistic or present bias on adopted model of TPB

In this study, the goodness-of-fit of the adopted model of TPB for medication adherence was verified with either optimistic bias or present bias as a moderating variable.

The results of comparing the goodness-of-fit between the adoption model and the models with moderating effects are given in Table 5. The χ^2^ value of the model with optimistic bias included as a moderating variable was 3.84 (*df* = 2, *p* = 0.147) and the goodness-of-fit indices were GFI = 0.996, NNFI = 0.974, CFI = 0.997, and RMSEA = 0.051 (0.000 ~ 0.128); the NNFI value was slightly better than the original adoption model, and the RMSEA value was less than 0.08, but much better than the original adoption model.

**Table 5:** Comparison of the goodness of fit with the moderating effects of optimistic and present bias

Model	χ^**2**^	*df*	GFI	NNFI	CFI	RMSEA (90% confidence interval)
Adopted model	3.18	1	0.996	0.970	0.997	0.078 (0.000 ~ 0.182)
Model with optimistic bias	3.84	2	0.996	0.974	0.997	0.051 (0.000 ~ 0.128)
Model with present bias	2.71	2	0.997	0.990	0.999	0.032 (0.000 ~ 0.115)

**Fig. 3:**
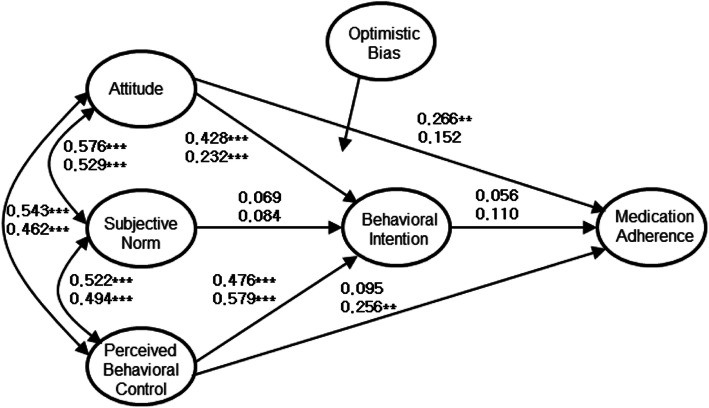
Path map of the model with a moderating effect of optimistic bias (^**^p < 0.01, ^***^p < 0.001; upper is for those with strong optimistic bias; lower is for those with weak optimistic bias

To clarify this point, the researcher analyzed the differences in two models with two samples to elucidate the moderating effects of optimistic bias on medication adherence and found a significant difference between the groups with strong and weak optimistic bias (*p* = 0.025). The NFI values of the unrestricted and restricted models also differed by .007, and the absolute value of the critical ratio in the unrestricted model was 2.25, exceeding 1.96, which indicates that the difference between the two groups was significant at the 0.05 level. These results suggested that there was a difference between the model with strong optimistic bias and the model with weak optimistic bias. As shown in Fig. 3, for groups with strong optimistic bias, only the attitudes → behavior path was significant, while for groups with weak optimistic bias, only the perceived behavioral control → behavior path was significant.

**Fig. 4:**
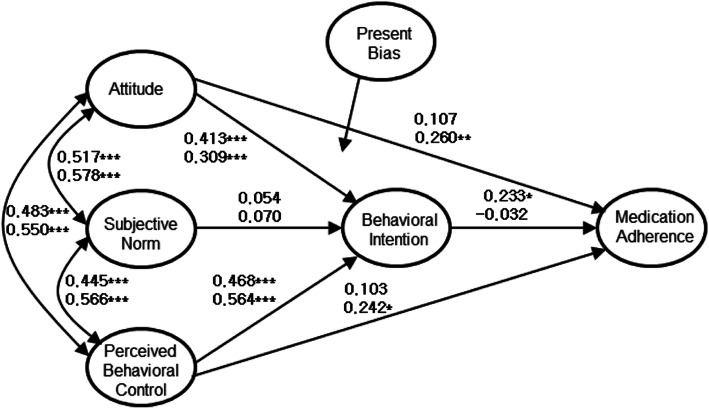
Path map of model with moderating effect of present bias (^*^p < 0.05, ^**^p < 0.01, ^***^p < 0.001; upper is for those with strong present bias, lower is for those with weak present bias)

The χ^2^ value of the model with present bias included as a moderating variable was 2.84 (*df* = 2, *p* = 0.258), and the goodness-of-fit indices for this model were GFI = 0.997, NNFI = 0.990, CFI = 0.999, and RMSEA = 0.032 (0.000 ~ 0.115); most of these values were better than those of the original adoption model, and in particular the RMSEA value was less than .05, the criterion for an excellent model. This result suggested that there was a significant difference between the model with strong present bias and that with weak present bias. As shown in Fig. 4, for groups with strong present bias, the proposed model was significant, while for groups with weak present bias, alternative model II was significant.

## Discussion

In situations in which prescription drugs play a central role in treatment, the patient’s medication adherence is as important as the medicinal effect of the drug. Therefore, if health professionals can improve the level of patients’ medication adherence, it could increase their likelihood of being treated properly. Thus, the present study investigated the psychological variables that may affect people’s general medication adherence and produced meaningful results.

The correlational analysis found that attitudes toward medication adherence and perceived behavioral control were closely related to behavioral intention of medication adherence, but the covariance with medication adherence was behavioral intention. As mentioned in the introduction, studies [20, 22] have found that among the variables of TPB, the behavior of perceived behavioral control accounts for the greatest variance in behavior, but in this study, the accountability of the attitude for medication adherence behavior was similar to perceived behavioral control in this regard. It was also shown that attitudes and perceived behavioral control could account for as much variance in medication adherence behavior as behavioral intention of medication adherence.

Thus, a model including the attitude → medication adherence and perceived behavioral control → medication adherence paths showed model conditions as good as the TPB model for medication adherence in this study. In this model, the path of behavioral intention → behavior, that is, medication adherence, was not significant. This may be because the attitude and perceived behavioral control were determinant variables for medication adherence in this model. Some studies [43] found low levels of accountability of behavioral intention in actual behavior. However, these results may vary depending on the TPB model for any particular behavior. All that can be concluded from this study is that Korean adults’ behavioral intention was significantly influenced by their attitudes toward it and toward perceived behavioral control. Although Hale, Household, and Greene [44] noted that the theory of reasoned action was developed because of “frustration with traditional attitude–behavior research, much of which found weak correlations between attitude measures and performance of volitional behaviors” (p. 259), the present study suggested that attitudes toward medication may play a direct role in ensuring that individuals take the medicine.

It was found that the subjective norms in an adopted model did not directly affect the behavioral intention for medication adherence in this study. Therefore, the indirect path of subjective norms → behavioral intention → medication adherence was also not significant. However, the subjective norms in this model shared a large amount of variance with attitude and perceived behavioral control, so it is believed that subjective norms would indirectly affect the behavior of medication adherence anyway, because people are influenced by the judgment of significant others [45] and subjective norms in this study were certain to interact with attitude toward medication adherence or perceived behavioral control.

Importantly, this study found that optimistic bias could moderate the TPB model for medication adherence. In other words, the TPB model for predicting the medication adherence of Korean adults showed significant differences in paths depending on the level of optimistic bias. This result suggested that optimistic bias could moderate the TPB model for health behaviors, particularly medication adherence behavior, beyond the results of previous studies [22, 23, 30] that found that optimistic bias makes individuals less likely to practice this health behavior. In the present study, for those with weak optimistic bias, attitudes in the TBP model did not directly affect medication adherence but did directly affect perceived behavioral control. On the other hand, for those with strong optimistic bias, perceived behavioral control did not directly affect medication adherence, but attitudes did directly affect it. In the TPB model of both groups, the direct path from behavioral intention to the behavior of medication adherence was not significant.

Therefore, it is difficult to apply the TPB model, proposed by Ajzen [8], where perceived behavioral control influenced the medication adherence of those with strong optimistic bias. To evaluate their medication adherence, one should thus identify whether they have a positive or negative attitude toward medication rather than examining other variables. In recent years, increasing self-efficacy is a common strategy for practicing health behaviors [46], but this study found that such a strategy does not work to improve the medication adherence of people with strong optimistic bias. Of course, because there were relatively close relationships among attitudes, subjective norms, and perceived behavioral control, while they may indirectly affect medication adherence, their influence in the model will not be significant. In conclusion, the present study suggested that such a strategy is likely to produce the desired effects only when applied to improve the medication adherence of those with weak optimistic bias. The higher the optimistic bias, the less likely the respondents were to think they will suffer any consequences for medication non-adherence. In other words, those with high optimistic bias were those who believed they would not have any adverse health consequences if they failed to take the medication as instructed. This result indicated that interventions should vary strategically depending on the level of optimistic bias, when intervening in attitudes, subjective norms or self-efficacy. For example, interventions targeting attitude may be more effective than interventions targeting other constructs in the model for those with high optimistic bias, while targeting perceived behavioral control may be more effective than interventions targeting other constructs in the model for those with low optimistic bias. It may be more efficient to educate or intervene by deciding whether to focus on having a positive attitude toward a health behavior or on enhancing self-efficacy according to the level of optimistic bias.

The present study found that the TPB model for medication adherence was also moderated by present bias. The TPB model for predicting the medication adherence of Korean adults was differentiated by whether there was delay discounting of expected medication effects. In the correlation analysis, the stronger the present bias, the less likely participants were to take medication, which extends research findings of the effects of delayed discounts on the results or effects of health risk behavior [32], smoking [33], and exercise [34] on medication adherence. Considering the moderating effects of present bias in the TPB model to predict medication adherence, the model proposed by Ajzen [8] was significant for those with strong present bias. On the other hand, for those with weak present bias, the model adopted in this study (alternative model II) was significant.

The moderating effect of present bias on the TPB model for medication adherence found in this study can be explained logically. The higher the level of present bias, the less likely respondents were to perceive the benefits of medication adherence, because they could not perceive the value of an effect that did not appear right away. That is why people with strong present bias are less likely to adhere to a medication if it does not show visible effects right away. It is predictable that attitude or perceived behavioral control does not directly affect medication adherence for those who recognize delay discounting on medication effects; thus such people should have a strong behavioral intention to take a given medicine. Therefore, medical or health professionals should focus on improving behavior intention to induce medication adherence in people with strong present bias. In this model, attitudes and perceived behavioral control had a significant impact on medication adherence behavior; thus, one such strategy is to have a positive attitude toward medicine and make behavioral control more perceptible to patients. In the case of people who were less likely to discount the effects of medication, if they had a positive attitude toward the medication or perceive themselves to have behavioral control, they were more likely to adhere to taking medication. In summary, while both groups showed significant positive attitudes toward medication and perceived behavioral control, it was important for those with strong present bias to have a definite behavioral intention. This result indicated where to strategically focus according to the level of delay discounting recognition, when intervening in attitudes, subjective norms or self-efficacy. For example, interventions targeting the attitude to change behavioral intention may be more effective than interventions targeting other constructs in the model for those with high present bias, while interventions targeting both attitude and perceived behavioral control may be equally effective for those with low present bias. It could be more efficient to intervene by deciding whether to focus on promoting a positive attitude toward a health behavior or by enhancing self-efficacy according to the level of delay discounting.

This study had several limitations. One limitation was the reliability of self-reporting MGLS, which measures participants’ medication adherence in this study. The internal consistency was 0.61 in the study of scale development [38] and 0.62 on this study. This scale is in the public domain and widely used and cited in peer-reviewed journals. However, reliability may be better when measured with the MMAS-8 (Morisky Medication Adherence Scale - 8) [47] which is widely used for medical purposes and can only be used with a license. Another limitation was that the study did not target patients with certain diseases; rather, it targeted the general population. Although this study was aimed at the general public because the health insurance system in Korea applies to all citizens, and almost all citizens take prescription medication for less serious diseases such as colds, the general population’s medication adherence may be too comprehensive in comparison to those of patients with specific diseases. There is also a general limitation of studies that rely on convenience sampling. Therefore, it is difficult to generalize the results of the study to the entire Korean population.

## Conclusions

In this study, the TPB model for adult medication adherence was validated, and a modified alternative model slightly modified from that proposed by Ajzen [8] was adopted. In addition, the TPB model adopted in this study was moderated by optimistic or present bias. This study was conducted with a Korean sample, and as the data were collected using convenience sampling, the sample was not representative of the total Korean population. Therefore, the results of the present study of the TPB model for medication adherence should be re-verified. Because no conclusion can be reached from a single study, the role of optimistic bias and present bias in medication adherence merits further research. Moreover, as there may be cultural differences in the roles of such variables [35], comparative cultural studies should be conducted on these topics. It is expected that the findings of this study will provide useful information for future research and for medical or health professionals who wish to improve the medication adherence of their patients.

## Data Availability

Any queries regarding the data used in this study may be directed to the corresponding author. The dataset used in the present study is available on reasonable request.

## Abbreviations

TPB: Theory of Planned Behavior
SPSS: Statistical Package for Social Sciences
AMOS: Analysis of Moment Structure
NNFI: (Non-normed Fit Index)
CFI: Comparative Fit Index
GFI: Goodness of Fit Index
RMSEA: Root Mean Square Error of Approximation
